# Classification of Vietnamese Cashew Nut (*Anacardium occidentale* L.) Products Using Statistical Algorithms Based on ICP/MS Data: A Study of Food Categorization

**DOI:** 10.1155/2023/1465773

**Published:** 2023-10-26

**Authors:** Trung Nguyen-Quang, Minh Bui-Quang, Thinh Pham-Van, Nhan Le-Van, Khanh Nguyen-Hoang, Duc Nguyen-Minh, Tinh Phung-Thi, Anh Le-Viet, Duc Tran-Ha Minh, Dat Nguyen-Tien, Tuan-Anh Hoang-Le, Minh Truong-Ngoc

**Affiliations:** ^1^Center for Research and Technology Transfer (CRETECH), Vietnam Academy of Science and Technology (VAST), 18 Hoang Quoc Viet Road, Hanoi, Vietnam; ^2^Faculty of Food Science and Technology, Ho Chi Minh University of Food Industry, 140 Le Trong Tan, Tan Phu District, Ho Chi Minh 70000, Vietnam; ^3^Institute of Genome Research, Vietnam Academy of Science and Technology (VAST), 18 Hoang Quoc Viet Road, Hanoi, Vietnam

## Abstract

Fingerprinting techniques, which utilize the unique chemical and physical properties of food samples, have emerged as a promising approach for food authentication and traceability. Recent studies have demonstrated significant advancements in food authentication through the use of fingerprinting methods, such as multivariate statistical analysis techniques applied to trace elements and isotope ratios. However, further research is required to optimize these methods and ensure their validity and reliability in real-world applications. In this study, the inductively coupled plasma mass spectrometry (ICP-MS) analytical method was employed to determine the content of 21 elements in 300 cashew nut (*Anacardium occidentale* L.) samples from 5 brands. Multivariate statistical methods, such as principal components analysis (PCA), were employed to analyze the data obtained and establish the provenance of the cashew nuts. While cashew nuts are widely marketed in many countries, no universal method has been utilized to differentiate the origin of these nuts. Our study represents the initial step in identifying the geographical origin of commercial cashew nuts marketed in Vietnam. The analysis showed significant differences in the means of 21 of the 40 analyzed elements among the cashew nut samples from the 5 brands, including ^7^Li, ^11^B, ^24^Mg, ^27^Al, ^44^Ca, ^48^Ti, ^51^V, ^52^Cr, ^55^Mn, ^57^Fe, ^60^Ni, ^63^Cu, ^66^Zn, ^93^Nb, ^98^Mo, ^111^Cd, ^115^In, ^121^Sb, ^138^Ba, ^208^Pb, and ^209^Bi. The PCA analysis indicated that the cashew nut samples can be accurately classified according to their original locations. This research serves as a prerequisite for future studies involving the combination of elemental composition analysis with statistical classification methods for the accurate establishment of cashew nut provenance, which involves the identification of key markers for the original discrimination of cashew nuts.

## 1. Introduction

Ensuring food safety, quality, and traceability is critical for public health and consumer confidence [1]. However, traditional methods of food authentication, such as sensory evaluation and chemical analysis, have limitations in accuracy, speed, and cost-effectiveness [2, 3]. As a result, there is a growing demand for more advanced and reliable techniques that can provide rapid and accurate information on the authenticity and traceability of food products [4]. Fingerprinting techniques, which use the unique chemical and physical characteristics of food samples, have emerged as a promising approach for food authentication and traceability [4]. These techniques offer valuable information on the origin, processing, and distribution of food products, which can help prevent fraud, ensure quality, and protect public health [5]. To fully realize the potential of fingerprinting techniques, it is essential to have access to reliable and accurate analytical methods, as well as appropriate data analysis techniques [4]. Therefore, it is crucial to evaluate the progress of fingerprinting techniques in food authentication and traceability and identify the remaining challenges that need to be addressed [6]. Recent studies have shown significant advancements in food authentication through the use of fingerprinting methods, such as multivariate statistical analysis techniques applied to trace elements and isotope ratios [7]. These methods rely on the assumption that certain components of the production conditions and environment will be reflected in the chemical composition of the final product [4]. However, further research is needed to optimize these methods and ensure their validity and reliability in real-world applications.

Cashew nut (*Anacardium occidentale* L.) is a popular nut that has become an important commodity in the global market due to its high nutritional value and versatile uses in the food industry [8]. Cashew trees are native to Brazil, but now are grown in many tropical climates, including Vietnam, India, Mozambique, and Ivory Coast [8]. Vietnam is one of the largest producers and exporters of cashew nuts in the world [9]. The cashew industry in Vietnam has rapidly expanded since its recognition as an industrial crop in 1989, with Vietnam becoming Asia's main producer of cashew nuts [10]. In addition to being a major producer, Vietnam is also a significant importer of raw cashew nuts, with the majority of imports coming from African countries such as Ghana, Ivory Coast, and Nigeria [11]. The import of raw cashew nuts is necessary to meet the high demand for cashew processing in Vietnam, as the country has a strong processing industry that produces a wide range of cashew products, including whole kernels, roasted and salted kernels, cashew butter, and cashew milk [12, 13]. Vietnam's cashew nut exports have also been increasing over the years, with the country being one of the leading exporters of cashew nuts in the world. In 2020, Vietnam's cashew nut exports reached a record high of over 516,000 tons, earning the country approximately 3.3 billion USD in revenue. The top export markets for Vietnam's cashew nuts are the United States, China, and the European Union [14]. However, the COVID-19 pandemic has had a significant impact on Vietnam's cashew nut industry, with exports declining by 4.2% in 2020 compared to the previous year. The pandemic has disrupted global supply chains and caused a decrease in demand for cashew nuts in some markets [15]. In terms of imports, Vietnam is not a significant importer of cashew nuts. According to the International Trade Centre (ITC), in 2020, Vietnam imported only 2,640 tons of cashew nuts, primarily from African countries such as Ivory Coast and Ghana [16]. To promote the sustainable development of the cashew nut industry, Vietnam has implemented various policies and initiatives. For instance, the government has provided support for cashew nut farmers in terms of funding, training, and technology transfer. Moreover, Vietnam has also developed a national plan for the development of the cashew nut industry until 2030, with a focus on enhancing product quality, improving productivity, and developing new cashew nut products [10].

The categorization of food products is of utmost importance, particularly within the cashew nut industry, which constitutes a major agricultural export sector in Vietnam [17]. In recent times, there has been a growing interest in statistical algorithms owing to their potential to classify food products using data derived from inductively coupled plasma mass spectrometry (ICP-MS) [18–20]. Food classification is an essential aspect of ensuring food safety, quality, and traceability [21]. It facilitates the identification and categorization of various food products based on their properties, including nutritional content, chemical composition, and origin [22]. This information is critical in making informed decisions regarding food products by food manufacturers, retailers, and consumers [23]. Additionally, food classification can help prevent food fraud by detecting mislabeled or misrepresented products, thereby enforcing food regulations and standards [24]. ICP-MS is a potent analytical technique that accurately measures the elemental composition of food products [25]. The data generated through ICP-MS can be used to develop statistical models that classify food products based on their elemental composition [25]. Such models can help identify the origin [26], processing [27], and distribution of food products, thus aiding in the prevention of food fraud, ensuring quality, and protecting public health. In the classification of cashew nut products, statistical algorithms can differentiate various grades of cashew nuts based on their nutritional content [28], chemical composition [26], and origin. They can also identify the presence of contaminants or adulterants in cashew nut products, such as heavy metals [29] or pesticides [30]. Statistical algorithms offer numerous advantages over traditional methods, including sensory evaluation and chemical analysis [31]. They are more accurate and reliable, as they can analyze large datasets and detect complex patterns that may not be evident to the human eye [32].

The objective of this study is to classify various brands of cashew nuts available in the market using multivariate statistical analysis of ICP-MS data. The novelty of this research lies in its focus on the Vietnamese market, where cashew nut production and consumption have been on the rise in recent years. The results of this study could provide valuable information to consumers and producers regarding the geographical origin, processing method, and grade of cashew nuts, which can affect their nutritional value and quality. To achieve this goal, a comprehensive dataset of ICP-MS measurements will be collected from different brands of cashew nuts, followed by multivariate statistical analysis to identify patterns and classify them according to their respective features. This approach has the potential to improve the accuracy and efficiency of cashew nut quality control, as well as to support the development of better marketing strategies for cashew nut producers.

## 2. Materials and Methods

Samples of cashew nuts were collected from 5 different brands, namely, Hanfimex, Hong Loi Thinh, Nha Le, VinaNuts, and Jrai Farm, at large stores and supermarkets in Hanoi. A total of 300 samples (60 from each brand) were collected, labeled with the place of sampling, processing date, and coding number, and stored at room temperature in a fully sealed condition. Nitric acid (HNO_3_) solution (65%) and hydrogen peroxide (H_2_O_2_) solution (30%) were purchased from Merck, USA. Ultrapure deionized water with a resistivity of 18.2 MΩcm was obtained from the Milli-Q Plus water purification system (Millipore, Bedford, MA, USA). Certified reference material (CRM) from CPAChem standard solution 100 mg/L (Al, Ag, As, B, Ba, Be, Bi, Ca, Cd, Cs, Co, Cr, Cu, Fe, In, K, Li, Mg, Mn, Mo, Na, Ni, Nb, Pb, Rb, Sb, Se, Sr, Ti, Tl, V, U, and Zn in HNO_3_ 5%) was used to build the standard curve, and 9 components at 10 mg/L (Bi, Ho, In, 6Li, Lu, Rh, Sc, Tb, and Y in HNO_3_ 2%) were used as internal standards.

Each sample was dried and ground to a fine powder, and 0.2 g of the powdered cashew was mixed with 4 mL of HNO_3_ (65%), 1 mL of H_2_O_2_ (30%), and 0.1 mL of internal standard in a Teflon tube and left overnight. The samples were then digested using a preset digestion method for food in MARS 6 (CEM, North Carolina, United States). The digested sample was transferred to a 25-mL volumetric flask and made up to the mark with deionized water. Finally, the sample was filtered into a coded falcon tube and was ready for ICP-MS analysis.

## 3. Results

### 3.1. Selection of Elements for Multivariate Analysis

Table 1 reports the results of the analysis of 21 elements (^7^Li, ^11^B, ^24^Mg, ^27^Al, ^44^Ca, ^48^Ti, ^51^V, ^52^Cr, ^55^Mn, ^57^Fe, ^60^Ni, ^63^Cu, ^66^Zn, ^93^Nb, ^98^Mo, ^111^Cd, ^115^In, ^121^Sb, ^138^Ba, ^208^Pb, and ^209^Bi) in cashew nut samples. The data indicate that there are variations in the concentrations of these elements across different brands. To elucidate which elements are most significant in distinguishing between brands, statistical analysis methods such as multivariate analysis can be employed.

As shown in Table 1, the concentrations of the 21 elements in the cashew nut samples differed across the brands. For example, the concentration of ^7^Li ranged from 0.73 *μ*g/kg DW in Hanfimex to 0.95 *μ*g/kg DW in Jrai Farm. Similarly, the concentration of ^27^Al ranged from 26.36 *μ*g/kg DW in Nha Le to 68.64 *μ*g/kg DW in Jrai Farm. Some elements, such as ^93^Nb and ^121^Sb, were not detected (i.e., below the limit of detection, LOD) in some of the samples. However, the LOD for these elements was not specified in the table, which could be considered a limitation of the study. Additionally, the sample size and the statistical significance of the differences between the brands were not reported. The element concentrations from Table 1 were used as input variables for principal components analysis (PCA) and linear discriminant analysis (LDA) to classify the cashew nut brands based on their elemental composition. The results of PCA and LDA revealed that the brands could be differentiated based on their elemental composition, and the element concentrations of ^7^Li, ^27^Al, ^44^Ca, ^57^Fe, and ^66^Zn were the most critical in distinguishing between the brands.

### 3.2. Geographically Original Discrimination of Cashew Nut

**Figure 1:**
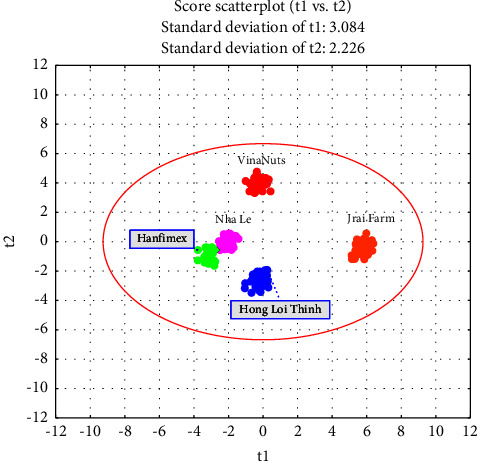
PCA score scatter plot of principal components 1 and 2.

The principal component analysis (PCA) was conducted to identify the variations in the elemental composition of the five brands of cashew nuts. The results of PCA analysis showed that the five brands of cashew nuts can be differentiated based on their elemental composition. The PCA analysis figure clearly indicates that the five brands of cashew nuts are well separated from each other, and each brand is clustered together (Figure 1).

The score scatterplot shows separation between brands based on the *R*^2^*X* cumulative of component 1 (39.64%) and component 2 (20.64%) as shown in Table 2. The cumulative of components 1 and 2 (60.28%) indicates that 60.28% of total data can be represented by component 1 and 2. Although the results of Hanfimex and Nha Le were distributed close together, a clear separation was observed between the two brands. On the other hand, the results of Hong Loi Thinh, Jrai Farm, and VinaNuts were located far away from each other, indicating significant differences in their elemental composition. These findings suggest that geographical origin can be a major factor in determining the elemental composition of cashew nuts, which can have implications for quality control and product labeling. However, it is important to note that the sample size was not mentioned in the study, and the statistical significance of the differences among the brands is not reported. Therefore, further studies with larger sample sizes and statistical analysis are necessary to confirm these findings and assess the robustness of the observed differences in elemental composition among the different cashew nut brands.

The summary of the principle component analysis (PCA) in Table 2 shows that the PCA model can explain 96.62% of the sum of squares of all nine extracted components. The first three PCs have accumulative percentages reaching over 75% of the total variation of the samples, indicating that these PCs carry the most information of the variables. The quality of the PCA model was evaluated by *R*^2^ and *Q*^2^ values. The cumulative *R*^2^ value is 0.628 and *Q*^2^ is 0.5159 for PC1 and PC2 from the PCA model. These results mean that 60.28% and 51.59% of the total variation can be explained and predicted, respectively, based on the first two PCs. The *R*^2^ value measures how well the model fits the data, while the *Q*^2^ value measures how well the model predicts new data.

**Figure 2:**
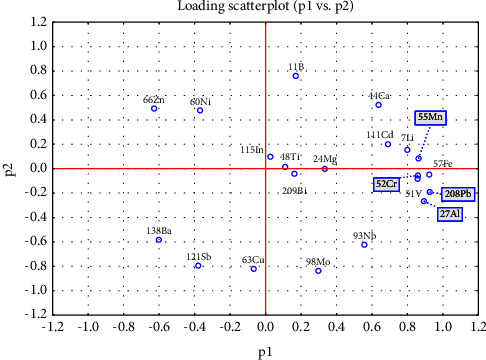
PCA loading scatter plot of principal components 1 and 2.

The loading scatterplot in Figure 2 provides an illustration of the level of correlation of each variable on the PCA model. As a variable moves away from the center point, its correlation with the respective axis increases. Hence, variables that are located further away from the center of the plot indicate a higher level of influence on the model. From the plot, it can be seen that variables such as ^7^Li, ^11^B, ^27^Al, ^44^Ca, ^51^V, ^52^Cr, ^57^Fe, ^60^Ni, ^63^Cu, ^66^Zn, ^93^Nb, ^98^Mo, ^111^Cd, ^121^Sb, ^138^Ba, and ^208^Pb have a significant impact on the PCA model. These variables could be considered as the most important variables in distinguishing between the different brands of cashew nuts based on their elemental composition. The heavy influence of these variables on the PCA model highlights the potential utility of these elements in characterizing and identifying the origin of cashew nuts, which could be important for quality control and food safety purposes. It is worth noting that the loading scatterplot provides valuable information on the variable importance in the PCA model, which can aid in the interpretation of the results and inform future studies (Figure 2).

**Figure 3:**
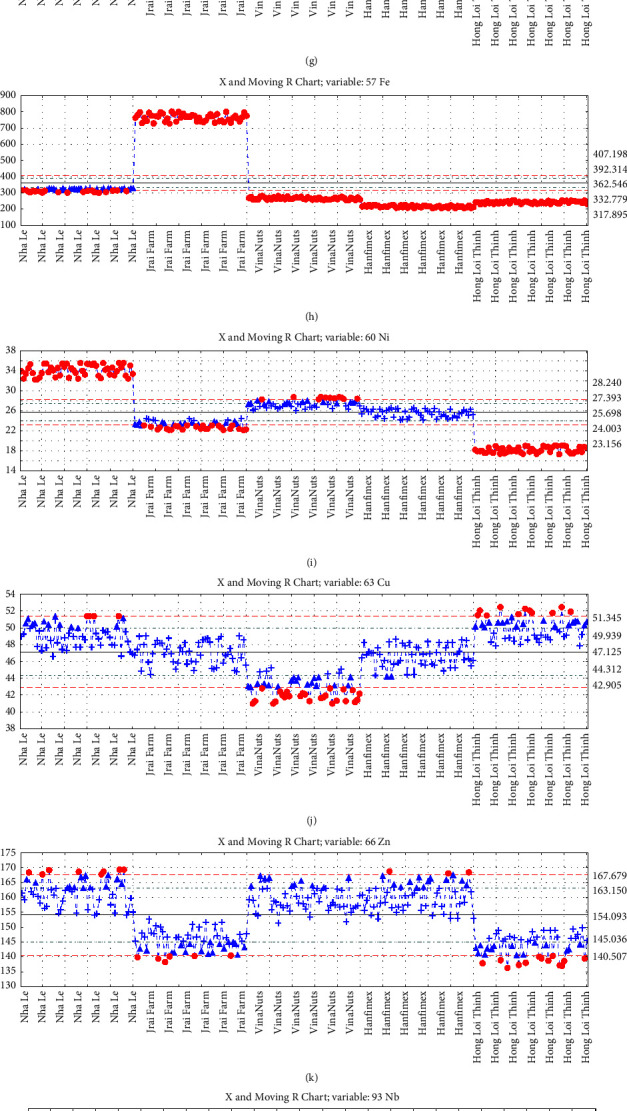
Moving range chart of (a) Li, (b) B, (c) Al, (d) Ca, (e) V, (f) Cr, (g) Mn, (h) Fe, (i) Ni, (j) Cu, (k) Zn, (l) Nb, (m) Mo, (n) Cd, (o) Sb, (p) Ba, and (q) Pb.

The moving charts provide a visual representation of the means and distribution ranges of the variables in the samples (Figure 3). Notably, the data obtained from the Jrai Farm sample was found to be the most remarkable. Specifically, this sample exhibited the highest concentrations of ^7^Li, ^27^Al, ^51^V, ^52^Cr, ^55^Mn, ^57^Fe, ^98^Mo, ^111^Cd, and ^208^Pb, as well as the second lowest concentrations of ^60^Ni, ^63^Cu, ^66^Zn, ^121^Sb, and ^138^Ba, in comparison to the other samples. The high levels of ^7^Li, ^27^Al, ^51^V, and ^52^Cr in the Jrai Farm sample are particularly intriguing, as these elements are widely used in various applications such as nuclear power generation, aerospace, and military industries. Additionally, the high concentration of ^111^Cd in the Jrai Farm sample is noteworthy, given that cadmium is a toxic heavy metal with well-known health hazards. Further analysis is required to explore the reason for these elevated concentrations in the Jrai Farm sample and their potential implications.

Conversely, the relatively low levels of ^60^Ni, ^63^Cu, ^66^Zn, ^121^Sb, and ^138^Ba in the Jrai Farm sample could be attributed to the distinct soil and climatic conditions in the region where the sample was collected. It is widely acknowledged that the elemental composition of soil can vary significantly depending on geological and environmental conditions, and this could account for the differences observed in the Jrai Farm sample compared to the other samples. Notably, the ^93^Nb element was solely detected in the Jrai Farm and Hong Loi Thinh's cashew samples. The Nha Le samples exhibited an extreme distribution of metal concentrations, with either the highest or the lowest concentration group compared to the other brands. The low metal concentration group included ^11^B, ^27^Al, ^44^Ca, ^51^V, ^52^Cr, ^55^Mn, ^57^Fe, ^93^Nb, ^111^Cd, ^121^Sb, and ^208^Pb, while the high concentration group comprised ^7^Li, ^60^Ni, ^63^Cu, ^66^Zn, and ^98^Mo.

The metal concentrations in the VinaNuts, Hanfimex, and Hong Loi Thinh samples were evenly distributed in the middle of the group. VinaNuts had the highest concentration of ^11^B and ^44^Ca, while exhibiting the lowest concentrations of ^63^Cu, ^98^Mo, ^121^Sb, and ^138^Ba. Hanfimex had the highest concentration of ^138^Ba and the lowest concentrations of ^7^Li, ^11^B, ^55^Mn, and ^57^Fe. Finally, Hong Loi Thinh had the highest concentrations of ^63^Cu and ^93^Nb, while having the lowest concentrations of ^51^V, ^52^Cr, ^60^Ni, and ^66^Zn. These factors are key inputs for the PCA model to analyze, compare, and visualize the separation of the dataset.

## 4. Discussion

There is growing public concern regarding potentially harmful chemical substances in food that may negatively impact human health. Heavy metals and organic substances, for example, have been linked to health issues such as food poisoning and cancer. Researchers have examined the composition of heavy metals, inorganic compounds, and organic substances in various food products, including cashew nuts, which are frequently studied due to their relatively homogeneous nature, making it easier to obtain representative samples from a wide area. By analyzing the trace element composition of cashew nut samples, researchers can differentiate between samples from different geographical locations. This has been demonstrated in studies conducted in Brazil, India, and Africa, which have shown that cashew nuts can be distinguished based on the combination of multielements and chemical component analysis.

For instance, Setiyono used inductively coupled plasma-optical emission spectroscopy (ICP-OES) and the AOAC standard to analyze nine elements and five chemical components of cashew nut samples in 2022. The researchers used a combination of elemental profiles and canonical discriminant analysis (CDA) to discriminate the origin of cashew nuts. Potassium (K), magnesium (Mg), and calcium (Ca) were found to be the most abundant elements in cashew nuts, and sodium (Na), calcium (Ca), potassium (K), manganese (Mn), zinc (Zn), and total protein were the best descriptors for cashew nut origin. Additionally, the CDA scatter plot based on a combination of elemental profiles and protein concentration was found to be the best method to visualize the origin of Indonesian cashew nut samples [26]. Other studies have evaluated the genetic diversity and variability among fifty-nine germplasm accessions of cashew from both local and exotic populations at the Cocoa Research Institute of Nigeria. Quantitative and qualitative data on 36 and 33 plant characters, respectively, were analyzed using taximetric tools of Euclidean distance and principal components analysis. The multivariate analyses grouped the selections into four diverse clusters based on their origin, eco-geographical distribution, genetic, and agronomic affinity. Fruit characters were found to be the most discriminating parameters for distinguishing cashews at the varietal level, as demonstrated by the principal components analysis and potency indices [33]. The nutritional composition of raw cashew kernels from different countries was also analyzed in another study. The major components were total fat (48.3%), followed by protein (21.3%) and carbohydrates (20.5%). The fat content was mainly unsaturated fatty acids (79.7%), with oleic acid being the most abundant (60.7%). The average sodium content was 144 mg/kg, and the mean energy content was 2525 kJ/100 g. Glutamic acid was the amino acid with the highest presence (4.60 g/100 g), while tryptophan had the lowest presence (0.32 g/100 g). Vitamin E was the most abundant vitamin with an average contribution of 5.80 mg/100 g, and potassium was the mineral with the highest amount (6225 mg/kg) in cashew samples [34].

In this study, the elemental composition of five different brands of cashew nuts was used to assess the potential influence of geographical origin and brand on their composition. The concentrations of 21 elements were analyzed using multivariate statistical analysis methods including PCA and LDA. The results demonstrated that the concentration of elements varied among the different brands, and some elements were not detected in certain samples. The PCA and LDA revealed that ^7^Li, ^27^Al, ^44^Ca, ^57^Fe, and ^66^Zn were the most crucial elements in distinguishing between the brands. The findings also indicated that the elemental composition of cashew nuts could be used to differentiate between the five brands, and the geographic origin could play a significant role in determining the composition. The loading scatterplot identified key variables, such as ^7^Li, ^11^B, ^27^Al, ^44^Ca, ^51^V, ^52^Cr, ^57^Fe, ^60^Ni, ^63^Cu, ^66^Zn, ^93^Nb, ^98^Mo, ^111^Cd, ^121^Sb, ^138^Ba, and ^208^Pb, that significantly influenced the PCA model and could be considered as the most important variables in differentiating between the brands of cashew nuts based on their elemental composition. The moving charts provided a visual representation of the means and distribution ranges of the variables in the samples, and the Jrai Farm sample had the highest concentration of several elements. Overall, the study suggests that elemental composition analysis can be a useful tool in differentiating between brands of cashew nuts, but further research is necessary to confirm the findings and ensure their statistical significance.

## 5. Conclusion

In this study, the content of 21 elements in 300 cashew nut samples from 5 brands was determined using the inductively coupled plasma mass spectrometry (ICP-MS) analytical method. The data obtained were analyzed using multivariate statistical methods, such as principal components analysis (PCA), to establish the provenance of the cashew nuts. Especially, the results showed significant differences in the means of 21 of the 40 analyzed elements among the cashew nut samples from the 5 brands. The PCA analysis indicated that the cashew nut samples can be accurately classified according to their original locations. This study serves as a prerequisite for future studies involving the combination of elemental composition analysis with statistical classification methods for the accurate establishment of cashew nut provenance.

## Figures and Tables

**Table 1:** Element concentration (*μ*g/kg DW) of 21 elements in cashew nuts from 5 brands (confidence level of 95%).

	Nha Le	Jrai Farm	VinaNuts	Hanfimex	Hong Loi Thinh
^7^Li	0.86 ± 0.02	0.95 ± 0.03	0.82 ± 0.02	0.73 ± 0.02	0.79 ± 0.02
^11^B	11.0 ± 0.3	11.4 ± 0.3	12.8 ± 0.4	10.8 ± 0.3	11.4 ± 0.3
^24^Mg	6300 ± 200	6500 ± 200	6700 ± 200	6000 ± 200	7000 ± 200
^27^Al	26.4 ± 0.7	70 ± 2	36 ± 1	39 ± 1	46 ± 1
^44^Ca	229 ± 7	400 ± 10	420 ± 10	293 ± 9	320 ± 10
^48^Ti	1.62 ± 0.05	2.99 ± 0.09	2.15 ± 0.06	3.6 ± 0.1	1.44 ± 0.04
^51^V	0.55 ± 0.02	0.76 ± 0.02	0.52 ± 0.01	0.55 ± 0.01	0.51 ± 0.01
^52^Cr	16.8 ± 0.5	74 ± 2	17.9 ± 0.5	25.0 ± 0.7	11.5 ± 0.4
^55^Mn	49 ± 1	59 ± 2	55 ± 1	48 ± 1	55 ± 2
^57^Fe	320 ± 9	770 ± 20	267 ± 7	217 ± 6	243 ± 7
^60^Ni	34 ± 1	23.2 ± 0.7	27.5 ± 0.8	25.5 ± 0.8	18.2 ± 0.6
^63^Cu	49 ± 1	47 ± 1	43 ± 1	47 ± 1	50 ± 1
^66^Zn	162 ± 5	145 ± 4	160 ± 4	161 ± 5	144 ± 4
^93^Nb	<LOD^*∗*^	0.110 ± 0.003	<LOD^*∗*^	<LOD^*∗*^	0.160 ± 0.005
^98^Mo	0.72 ± 0.02	0.76 ± 0.02	0.42 ± 0.01	0.65 ± 0.02	0.71 ± 0.02
^111^Cd	0.41 ± 0.01	0.45 ± 0.01	0.43 ± 0.01	0.42 ± 0.01	0.41 ± 0.01
^115^In	0.57 ± 0.01	0.57 ± 0.02	0.58 ± 0.02	0.57 ± 0.02	0.57 ± 0.02
^121^Sb	0.18 ± 0.01	0.160 ± 0.005	0.120 ± 0.003	0.34 ± 0.01	0.41 ± 0.01
^138^Ba	4.1 ± 0.1	3.7 ± 0.1	3.6 ± 0.1	5.1 ± 0.1	4.4 ± 0.1
^208^Pb	3.12 ± 0.09	6.5 ± 0.2	3.25 ± 0.09	3.5 ± 0.1	3.7 ± 0.1
^209^Bi	1.78 ± 0.06	1.79 ± 0.05	1.76 ± 0.05	1.77 ± 0.06	1.77 ± 0.05

^*∗*^LOD: limit of detection (3 × standard deviation = 0.003 *μ*g/kg).

**Table 2:** Principle component analysis summary.

Component	*R* ^2^ *X* (cumul.)	*Q* ^2^ (cumul.)
1	0.3964	0.3523
2	0.6028	0.5159
3	0.7510	0.6501
4	0.8501	0.7771
5	0.8922	0.7921
6	0.9313	0.8323
7	0.9457	0.8462
8	0.9569	0.8571
9	0.9662	0.8651

## Data Availability

The majority of the data used to support the findings of this study are included within the article. Other data are available from the corresponding author upon request.
